# Elucidation of flavonoids from potent Iranian *Scutellaria* species against Influenza A (H1N1) virus

**DOI:** 10.22038/IJBMS.2022.66255.14553

**Published:** 2023-01

**Authors:** Mostafa Pirali Hamedani, Mahtab Ahmad Kashi, Saied Goodarzi, Hadiseh Shokouhi, Mahdi Shafiee Ardestani, Abbas Hadjiakhoondi, Morteza Pirali Hamedani, Mohammad Hosein Ghahremani, Parvaneh Mehrbod, Zahra Tofighi

**Affiliations:** 1 Department of Pharmacognosy, Faculty of Pharmacy, Tehran University of Medical Sciences, Tehran, Iran; 2 Medicinal Plants Research Center, Faculty of Pharmacy, Tehran University of Medical Sciences, Tehran, Iran; 3 Influenza and Respiratory Viruses Department, Pasteur Institute of Iran, Tehran, Iran; 4 Department of Radiopharmacy, Faculty of Pharmacy, Tehran University of Medical Sciences, Tehran, Iran; 5 Department of Medicinal Chemistry, Faculty of Pharmacy, Tehran University of Medical Sciences, Tehran, Iran; 6 Department of Pharmacology and Toxicology, Faculty of Pharmacy, Tehran University of Medical Sciences, Tehran, Iran

**Keywords:** Antiviral, Flavonoids, Folin-ciocalteau reagent, Influenza A virus, MTT assay, *Scutellaria*

## Abstract

**Objective(s):**

Influenza A virus (IAV) is a contagious illness. Different species of *Scutellaria* genus are used as a traditional remedy to reduce influenza symptoms. This study aimed to investigate the anti-influenza capacity of several species of Iranian Scutellaria and identify active compounds of the most potent species for the first time.

**Materials and Methods:**

Some Iranian species of *Scutellaria* were collected from different regions of Iran, including *S. pinnatifida* with *mucida*, *viridis*, and *alpina* subspecies; *S. tournefortii*; *S. tomentosa*; *S. persica*. They were fractionated to chloroform and methanol. The total phenols and flavonoids of samples were examined by the folin-ciocalteau and aluminum-flavonoid complex methods, respectively. The 50% cytotoxic concentrations (CC_50_) on MDCK cells and non-cytotoxic concentrations (NCTC) were determined by MTT assay. The percentage of cell protection against IAV and their effect on virus titer were investigated in pre-, post-, and co-penetration treatment groups. Phytochemicals of the most effective species were isolated by various chromatographic methods and identified by different spectroscopic methods.

**Results:**

Methanol fraction of *S. pinnatifida* subsp. *viridis* demonstrated the highest amounts of flavonoid content and best activity against influenza A virus in all combination treatments, which reduced the virus titer by 5 logs with no cytotoxicity. Kaempferol-3-*O*-glucoside, quercetin-3-*O*-glucoside, apigenin-4′-methoxy-7-*O*-glucoside, luteolin, and luteolin-7-*O*-glucoside were purified and identified from this species.

**Conclusion:**

*Scutellaria pinnatifida* subsp. *viridis* can be introduced as a source of flavonoids with acceptable anti-influenza activity. *S. tomentosa* also showed potent antiviral effects and is a candidate for elucidation in further studies.

## Introduction

Influenza virus can cause dangerous illnesses due to the high potential of mutations (antigenic drifts and shifts) in viral genes, and flu infection can result in hospitalization and death (1). H1N1 is a subtype of influenza A virus (IAV) that can cause upper and lower respiratory tract infections. Symptoms like fever, chills, cough, shortness of breath, myalgia, and gastrointestinal symptoms like abdominal pain and vomiting have been reported in some cases (2). Currently, two classes of influenza antiviral drugs are available in the market; first, M2 proton channel blockers (amantadine and rimantadine) and second, neuraminidase inhibitors (oseltamivir, zanamivir, etc.) (3), however, because of drug resistance concerns, it is crucial to discover and improve new compounds (4). 

Medicinal plants have represented great potential for antiviral activity against different types of viruses (5). Numerous studies have shown that natural compounds derived from herbal sources can be used as lead compounds for novel drugs. Anti-influenza studies have been done using different plants, and some of those like *Sambucus nigra, Kaempferia parviflora, Curcuma longa, Ephedra sinica, Eleutherococcus senticosus, Glycyrrhiza glabra, Brassica juncea, *and* Scutellaria baicalensis *showed significant antiviral activity against influenza virus affecting different pathways (6-10).

*Scutellaria* (Lamiaceae), with the common name of “Skullcap” in English and “Kolah-khoodi” in Persian, includes more than 350 species in several regions like East Asia, Europe, and North America. It is a traditional herbal medicine with a long history of treating various diseases like diarrhea, hypertension, insomnia and respiratory infection for thousands of years (11, 12). The Iranian flora includes more than 20 species distributed in different regions (13).

In recent years different activities of the *Scutellaria* genus have been studied. Anti-tumor action against breast and colon cancer, an anti-oxidant activity that can decrease cell damage by inhibiting oxidative stress (14), anti-inflammatory activity via different pathways (15), antibacterial and antiviral effects against various pathogens, effects on vascular diseases (cerebrovascular and cardiovascular) (16), and some other pharmacological activities like hepatoprotective and neuroprotective have been reported (15). Thus, modern pharmacology has proved some of the traditional usages of these plants and confirmed the value and potential of *Scutellaria* species. The presence of lots of flavonoids and other compounds seems to be the main reason for these effects (17).

One of the major groups of secondary metabolites which plants produce is phenolic compounds. The health benefits of fruits and vegetables are related to their high phenolic content (18). One of the beneficial effects of phenolic compound derivatives is their antiviral effects (19). About half of the phenolic compounds are presented in the flavonoids group, which are responsible for some plant activities. For example, attracting insects through attractive colors is probably due to the presence of flavones, flavonols and anthocyanidins, which leads to their pollination by insects. Also, catechins and some flavonols are responsible for defending plants against harmful insects (20). Flavonoids play a fundamental role in plant development and survival and can be used to prevent and treat some diseases in humans. Some of these properties are anti-atherosclerotic, anti-inflammatory, anti-thrombogenic, anti-tumor, anti-osteoporotic, antiviral, antibacterial and anti-fungal effects. Also, flavonoids are known as anti-oxidant, hepato-protectant, cardio-protectant, anti-diabetic, and anxiolytic compounds (21). In this study, the phenolic and flavonoid contents and antiviral activity of some Iranian *Scutellaria* species were studied to find the potent species and also the isolation and identification of phytochemicals of the potent species.

## Materials and Methods

***Plant materials***

Four species of *Scutellaria* were collected from different regions of Iran. The identity of three species was verified by the botanist Dr Y Ajani with voucher specimen and registered in the Herbarium of the Faculty of Pharmacy, Tehran University of Medical Sciences, Tehran, Iran. The voucher specimen of the other species was registered in the Herbarium of Institute of Medical Plants, Karaj, Iran and identified by Dr M Ghorbani Nohooji (Table 1). 

***Extraction and fractionation***

All collected species of *Scutellaria* were dried in the shade at room temperature. When the final moisture level of the plants was minimized, the plants were pulverized and the extraction process was accomplished with methanol: water (8:2) solvent by maceration method (total extract). Then, the extracts were fractionated by chloroform (chloroform fractions) and the residues were named methanol fractions.

***Total phenol content assay***

The Folin-ciocalteau method was selected for determining the total phenol content of all fractions of *Scutellaria* species. The concentration of the samples was equal to 500 μg/ml, diluted with methanol. Folin-ciocalteau reagent (1.5 ml) (1:10 diluted with deionized water) was mixed with 200 μl of each sample. After 5 min, 1.5 ml of sodium bicarbonate (6 g/l was dissolved in distilled water) was added. The absorption of these solutions was measured after 90 min incubation in a dark place at room temperature. Followed by that, the absorbance was measured at 725 nm by the spectrophotometer. Methanol was considered as control. The calibration curve of Gallic acid (0, 25, 50, 100 mg/ml) was plotted as a standard curve. The total phenol content of the fractions was expressed as mg of Gallic acid per gram of fraction (22).

***Total flavonoid content assay***

Among several methods for determining the total flavonoid content, the aluminum-flavonoid complexing method was selected. The volume of 1 ml of each sample (100 μg/ml) was diluted with 4 ml distilled water, and then 0.3 ml sodium nitrite 5% w/v was added. After 5 min, 0.3 ml of aluminum chloride 10% w/v was added. Five minutes later, 2 ml sodium hydroxide 4% w/v was added and the final volume of the solution reached 10 ml with distilled water. The absorbance of samples was measured at 510 nm after 30 min. Preparing the blank sample was the same as other samples, except there was no analyte and aluminum chloride in the blank sample and methanol was substituted instead (23).

Catechin (25, 75, 125, 150, 500 μg/ml) was used as positive control and the results of the total flavonoid content of *Scutellaria* species were reported as mg of catechin per gram of fraction.

***Cell culture and influenza virus multiplication***

The cell line of Madin Darby Canine Kidney (MDCK) was used to propagate the influenza virus. MDCK cells were maintained in Dulbecco’s Modified Eagle’s Medium (DMEM) (Mediatech Cellgro, USA) plus 10% Fetal Bovine Serum (FBS) (PAA, Austria) and 100 μg/ml of Penicillin and 100 μg/ml of streptomycin (Pen/Strep 1% (Sigma Co.)). IAV [A/Puerto Rico/8/1934 (H1N1) (ATCC VR-1469™)] was provided by the Influenza and Respiratory Viruses Department, Pasteur Institute of Iran. The virus infectivity dose as the 50% cell culture infectious dose (CCID_50_) was determined using the Karber method (24, 25).

***Cytotoxicity assay***

All the fractions were exposed to the MDCK cells at different concentrations (3.125, 1.562, 0.781, 0.390, 0.195, 0.097, 0.048, 0.024, 0.012, 0.006, and 0.003 mg/ml) and incubated at 37°C, 5% CO_2_ incubator for 48 hr. Then, the culture medium was removed, and 100 μl of MTT 1X [3-(4,5-dimethyl-2-thiazolyl)-2,5-diphenyl-2H-tetrazolium bromide; Sigma, USA] was added to the cells and incubated for 3 to 4 hr in the dark. Following this time, 100 μl of DMSO was added to dissolve the precipitates of formazan in purple color. A microplate reader (BioTek EL 800, US) was set to record the optical density (OD) at 540 nm, and the cell viability was measured based on the following formula: 

Cell viability = (mean OD of treated cells/mean OD of control cells) × 100

The concentration that caused visible morphological changes in 50% of the cells was defined as CC_50_. MTT data were analyzed with SPSS software, and related graphs were plotted to find NCTC for each sample. Also, the selectivity index (SI) that represents the safety of each extract was calculated by the division of CC_50_ to NCTC (26). 

***Antiviral assay***

Three combination treatments of co-, pre-, and post-penetration were evaluated to determine the antiviral ability of the fractions. For the co-penetration treatment, 100TCID_50_ (Tissue Culture Infectious Dose 50%, which is defined as the dilution of virus required to infect 50% of a given cell culture) of the virus was mixed with NCTCs for 30 min and then exposed to MDCK cells in 96-well plates and incubated for 1 hr. For the other two procedures, the virus was added to the cells after (pre-) and before (post-) the fractions following 1 hr incubation. Finally, unabsorbed viruses were replaced by Tosyl phenylalanyl chloromethyl ketone (TPCK)-containing medium (1 µg/ml), and all the plates were incubated for 48 hrs. The MTT assay was performed to measure cellular protection against virus infection in an antiviral assay. Concurrently, the hemagglutination assay (HA) was used to determine the virus titer in the supernatants (25).

The percentage of protection was calculated by SPSS using MTT data of cell viabilities after 48 hrs of exposure by the following formula: 

Percentage of protection= [(ODT) V − (ODC) V] / [(ODC)M − (ODC) V] × 100

Where (ODT) V, (ODC) V and (ODC) M represent the absorbance of the treated sample, the virus-infected control (no compound) and the negative control (mock) (27). 

***Hemagglutination assay (HA)***

HA assay was used to determine the virus titer. Briefly, 100 μl of each supernatant was added to a 96-well U-shaped microplate, and 2-fold serial dilutions were prepared using 50 μl PBS in each well. Then, chicken RBC (cRBC) 1% was added to all wells and stayed at room temperature for 1 hr. The formation of clots showed the absence of the virus. HA units were the reciprocal of the highest dilution giving complete agglutination with cRBC (28).

***Hemagglutination inhibition assay (HI)***

Two-fold serial dilutions from CC_50_ of each fraction were prepared to investigate the inhibitory outcome of the fractions on the hemagglutinin (HA) glycoprotein activity and then 4HA units of the virus particles were added to each well. Forty-five min of pre-incubation at room temperature was needed. Then, cRBC was added to each well. After 1 hr, the physical interaction between the fractions and virus surface HA glycoprotein was measured. 

***Statistical analysis***

The results of cell protection and the logarithm of HA decrement were analyzed by one-way ANOVA and Fisher’s LSD *post hoc* test using IBM SPSS Statistics 23 (*P*-value <0.05). 

***Separation, isolation, and identification***

According to the results of total phenol, flavonoid content, and antiviral assays, *S.* *pinnatifida *subsp. *viridis* was considered as the potent species collected from the Zarabad region, Qazvin province, Iran. Therefore, this species was selected for the phytochemical analysis. First, Diaion HP-20 resin was used for the purification of polyphenols. It adsorbed 10 g of the total extract. Non-phenolic compounds were washed out with distilled water, and phenolic compounds were eluted by methanol (100%), which was named methanol fraction.

Separation and isolation of the methanol fraction of *S.* *pinnatifida* subsp. *viridis* was as follows: 3 g of methanol fraction (A) was loaded on a reversed-phase silica gel column (3 × 30 cm) and eluted with water: methanol (8:2) to methanol 100%. Compound **1** (21.2 mg) was elucidated from sub-fraction No. 16. Sub-fraction No. 8 (A8; 271.1 mg) was selected for the next stage. Sub-fraction A8 was loaded on reversed-phase silica gel (2 × 35 cm) and washed out by water: methanol (8:2) to pure methanol. According to the TLC investigation, sub-fraction A8f (3.2 mg) was a pure compound (Compound **2**).

Sub-fraction A4 (165.7 mg) and A28 (48 mg) were loaded on Sephadex LH-20 (1.5 cm × 60 cm), respectively. The columns were rinsed with methanol (100%). Compound **3** (28.1 mg) and compound **4** (2 mg) appeared as one spot on TLC paper under UV radiation and by coloring with an anisaldehyde solution reagent. A4b (48 mg) was loaded on a reversed-phase silica gel column (3 × 30 cm) with gradient solvent from pure water to pure methanol and compound **5** (3.4 mg) was elucidated as A4b (15 mg). 

After separation, isolation and purification steps, the compounds were identified by different spectroscopic methods, including UV, ^1^H-NMR, and ^13^C-NMR. 

## Results

***Total phenol content***

The total phenol content of different species of *Scutellaria* was determined by the Folin-ciocalteau method and reported based on the Gallic acid equivalent.

***Total flavonoid content***

The presence of flavonoids in fractions caused an orange color in the sample due to the formation of an aluminum-flavonoid complex. Data were reported as mg of catechin per gram of fraction. The total phenol and flavonoid content of each species of *Scutellaria* is summarized in Table 2.

***Cytotoxicity and antiviral activity of the compounds***

This study tested the efficacy of chloroform and methanol fractions of the selected traditional Iranian plants against IAV. The cytotoxicity of the fractions was evaluated. The CC_50_ and NCTC of the fractions were defined before the antiviral assay. The ability of different fractions to reduce viral titer and the viral cytopathic effects varied as determined by the HA and MTT assays. The cytotoxicity profile of the fractions used in this study is listed in Table 3.

Among the chloroform fractions,* S. tournefortii *(Gorgan) and *S. pinnatifida *subsp. *mucida* (Barajin) showed the highest (771.74±2.89 µg/ml) and the lowest (37.07±0.71 µg/ml) CC_50_ values, respectively. Among methanol fractions, the highest and the lowest CC_50_ values were obtained with *S. tournefortii *(Ramsar) (6157.12±1.02 µg/ml) and *S. tournefortii* (Gorgan) (3.07±0.19 µg/ml), respectively.

**Figure 1:**
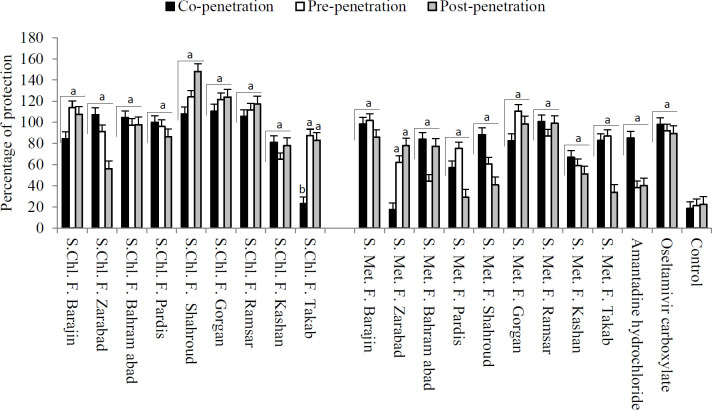
Percentage of cell protection of different fractions of *Scutellaria* species in combination treatment against influenza A virus (S: *Scutellaria*, Chl: Chloroform, Met: Methanol, F: Fraction. Barajin: *S. pinnatifida* subsp. *mucida*, Zarabad: *S. pinnatifida* subsp. *viridis*, Bahram abad:* S. pinnatifida* subsp. *viridis*, Pardis: *S. pinnatifida* subsp. *viridis*, Shahroud:* S. pinnatifida* subsp. *alpina*, Gorgan: *S. tournefortii*, Ramsar: *S. tournefortii*, Kashan: *S. tomentosa*, Takab: *S. persica*, a: Significant difference between treatment group and control with *P*<0.01, b: Significant difference between treatment group and control with *P*<0.05)

The percentage of cell protection was a factor in demonstrating cell viability in the presence of the influenza virus. All samples, except the methanol fraction of *S. pinnatifida* subsp. *viridis* (Zarabad) (17.36±3.72 %), and chloroform fraction of *S. persica* (Takab) (23.16±6.29 %) showed optical absorption over 0.5 in co-penetration treatment. In pre-penetration treatment, all samples demonstrated optical absorption above 0.5. In post-penetration treatment, methanol fraction of *S. pinnatifida* subsp. *viridis* (Kamard) (29.17±1.53 %) and *S. percisa* (Takab) (33.78±3.84 %) showed optical absorption over 0.5, which represents cell protection. Oseltamivir as positive control had optical absorption over 1.00 in all treatments. Statistical analyses demonstrated significant differences between pre-, co-, and post-penetration treatment groups and the control group in cell viability percentage with *P*-value<0.05 (Figure 1). So, the samples with a cell protection percentage over 40% were suitable candidates for the anti-influenza effect. Therefore, to obtain the potent extracts or fractions of *Scutellaria* with an anti-influenza effect, the percentage of cell protection was examined along with the hemagglutination. 

**Figure 2:**
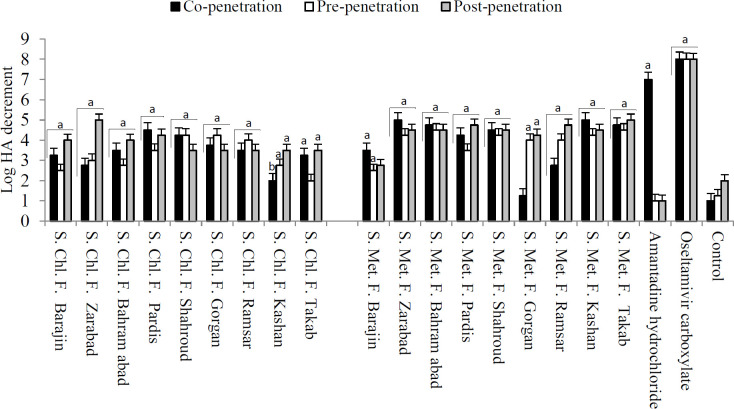
Anti-influenza A effects of different fractions of *Scutellaria* species (Log HA: Logarithm of Hemagglutinin, S: *Scutellaria*, Chl: Chloroform, Met: Methanol, F: Fraction. Barajin: *S. pinnatifida* subsp. *mucida*, Zarabad: *S. pinnatifida* subsp. *viridis*, Bahram abad: *S. pinnatifida* subsp. *viridis*, Pardis: *S. pinnatifida* subsp. *viridis*, Shahroud: *S. pinnatifida *subsp. *alpina*, Gorgan: *S. tournefortii,* Ramsar: *S. tournefortii*, Kashan: *S. tomentosa*, Takab: *S. persica*, a: Significant difference between treatment group and control with *P*<0.01, b: Significant difference between treatment group and control with *P*<0.05)

The fractions could decrease viral hemagglutinin activity, and HA logarithm decrement demonstrated the potency of the extracts or fractions. Regarding HA results, in co-penetration treatment, methanol fraction of Zarabad (5.00±0.82) and *S. tomentosa* (Kashan) (5.00±0.82) reduced the virus titer by 5 logs, and amantadine reduced the virus titer by 7 logs. In pre-penetration treatment, the methanol fraction of *S. pinnatifida* subsp. *viridis* (Bahram abad) and Takab reduced the virus titer by 4.5 logs. In post-penetration treatment, the methanol fraction of Takab, and chloroform fraction of Zarabad reduced the virus titer by 5 logs. Oseltamivir reduced virus titers by 8 logs in all treatments. Statistical analyses demonstrated significant differences between pre-, co-, and post-penetration treatment groups and the control group in HA-log decrement with *P*-value < 0.05 (Figure 2).

***Phytochemical study***

Ultraviolet and ^1^H-NMR spectra of the compounds were investigated and demonstrated as follows:

**Compound 1:** Kaempferol-3-*O*-glucoside or Astragalin, brownish yellow color, UV λ_max_-nm MeOH: 265, 348 nm, ^1^H-NMR (500 MHz, DMSO-*d*_6_): δ 8.08 (2H, *dd*, *J*=8, 2 Hz, H-2′, H-6′), 7.60 (2H, *dd*, *J*=8, 2 Hz, H-3′, H-5′), 7.06 (1H, *d*, *J*=2 Hz, H-8), 7.01 (1H, *d*, *J*=2 Hz, H-6), 5.18 (1H, *d*, *J*=7.7 Hz, anomeric hydrogen of glucose), 3.5-4.5 (hydrogens of glucose) (Suppl. Figure 1).

**Compound 2:** Quercetin-3-*O*-glucoside, brownish yellow color, UV λ_max_-nm MeOH: 275, 335 nm, ^1^H-NMR (500MHz, DMSO-*d*_6_): δ 7.50 (2H, *d*, *J*=2 Hz, H-2′, H-6′), 6.93 (1H, *d*, *J*=8 Hz, H-5′), 6.72 (1H, *d*, *J*=2 Hz, H-8), 6.64 (1H, *d*, *J*=2 Hz, H-6), 4.94 (1H, *d*, *J*=7.5 Hz, anomeric hydrogen of glucose), 3.5-4.5 (hydrogens of glucose) (Suppl. Figure 2).

**Compound** **3:** Luteolin-7-*O*-glucoside, dark yellow color UV λ_max_-nm MeOH: 255, 349 nm, ^1^H-NMR (500 MHz, DMSO-*d*_6_): δ 7.43 (2H, *dd*, *J*=8, 2 Hz, H-2′, H-6′), 6.90 (2H, *d*, *J*=8 Hz, H-5′), 6.80 (1H, *d*, *J*=2 Hz, H-8), 6.74 (1H, *s*, H-3), 6.45 (1H, *d*, *J*=2 Hz, H-6), 5.19 (1H, *d*, *J*=7.5 Hz, anomeric hydrogen of glucose), 3.5-4.5 (hydrogens of glucose) (Suppl. Figure 3). 

**Compound** **4:** Luteolin, yellow color, UV λ_max_-nm MeOH: 264, 357 nm, ^1^H-NMR (500 MHz, DMSO-d_6_): δ 7.40 (1H, *dd*, *J*=8, 2 Hz, H-6′), 7.36 (1H, *d*, *J*=2 Hz, H-2′), 6.82 (1H, *d*, *J*=8 Hz, H-5′), 6.60 (1H, *s*, H-3), 6.39 (1H, *d*, *J*=2 Hz, H-8), 6.13 (1H, *d*, *J*=2 Hz, H-6) (Suppl. Figure 4).

**Figure 3:**
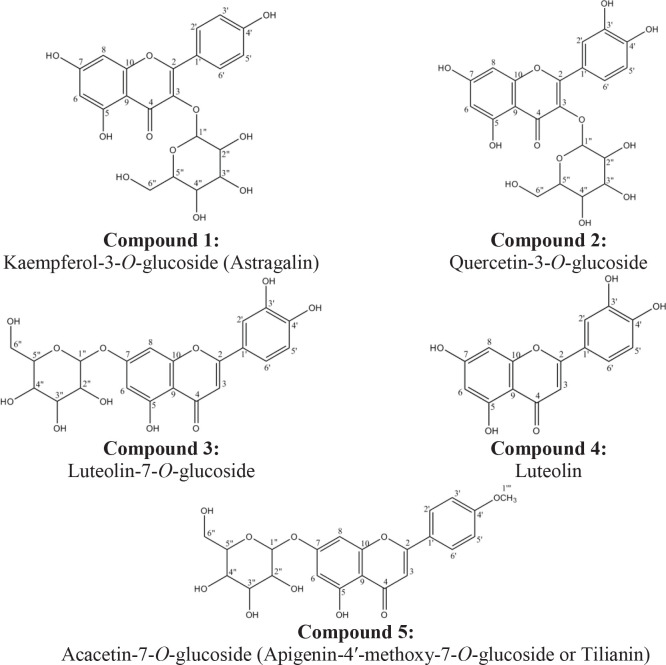
Elucidated compounds from *Scutellaria pinnatifida* subsp. *viridis*

**Compound** **5:** Tilianin or Acacetin-7-*O*-glucoside or Apigenin-4′-methoxy-7-*O*-glucoside, pale yellow color, UV λ_max_-nm MeOH: 272, 341 nm, ^1^H-NMR (500 MHz, DMSO-*d*_6_): δ 7.45 (2H, *dd*, *J*=8, 2 Hz, H-2′, H-6′), 7.06 (1H, *s*, H-3), 6.90 (2H, *dd*, *J*=8 , 2 Hz, H-3′, H-5′), δ 6.67 ppm (1H, *d*, *J*=2 Hz, H-8), δ 6.36 ppm (1H, *d*, *J*=2 Hz, H-6), δ 5.18 ppm (1H, *d*, *J*=7.7 Hz, anomeric hydrogen of glucose), δ 3.5-4.5 ppm (hydrogens of glucose), δ 3.81 ppm (3H, *s*, 4′-OCH3) (Suppl. Figure 5). The structures of the flavonoid compounds are demonstrated in Figure 3.

**Table 1:** Profile of location of various *Scutellaria* species from Iran

**Scientific name**	**Voucher No.**	**Elevation**	**Location**	**Code**
*Scutellaria pinnatifida* subsp.* mucida *(Stapf) Rech.f.	7043-TEH^**^	1600 m^*^	Barajin, Qazvin Province	Barajin
*Scutellaria pinnatifida* subsp.* viridis *(Boiss.) Rech.f.	7040-TEH	1650 m	Zarabad, Qazvin Province	Zarabad
*Scutellaria pinnatifida* subsp.* viridis *(Boiss.) Rech.f.	7042-TEH	1800 m	Bahram abad, Qazvin Province	Bahram abad
*Scutellaria pinnatifida* subsp.* viridis *(Boiss.) Rech.f.	7039-TEH	1500 m	Kamard, Tehran Province	Kamard
*Scutellaria pinnatifida* subsp.* alpina *(Bornn.) Rech.f.	7117-TEH	2300 m	Shahroud, Semnan province	Shahroud
*Scutellaria tournefortii *Benth.	7037-TEH	550 m	Galikesh, Golestan Province	Gorgan
*Scutellaria tournefortii *Benth.	7046-TEH	500 m	Ramsar, Mazandaran Province	Ramsar
*Scutellaria tomentosa *Bertol.	7116-TEH	2000 m	Kashan, Isfahan Province	Kashan
*Scutellaria persica *Bornm.	7078-IMPH^***^	2500 m	Takab, West Azarbaijan Province	Takab

*m: meter; **TEH: Herbarium of Faculty of Pharmacy, Tehran University of Medical Sciences, Tehran, Iran; ***IMPH: Herbarium of Iranian Institute of Medicinal Plants, Karaj, Iran

**Table 2:** Total phenol and flavonoid contents of different species of *Scutellaria*

*Scutellaria* samples^*^		Total Phenol Contents (mg gallic acid/ g fraction)	Total flavonoid Contents (mg catechin/ g fraction)
Chloroform	Barajin	224.4±1.6	41.9±4.6
	Zarabad	160.6±5. 9	226.7±6.3
	Bahram abad	118.6±5.9	226.4±5.7
	Tehran	95.2±2.5	199.9±2.0
	Shahroud	117.1±7.5	168.1±3.8
	Gorgan	89.0±2.3	54.7±8.9
	Ramsar	89.7±0.6	88.9±9.5
	Kashan	158.4±4.2	164.1±0.8
	Takab	141.9±4.1	121.1±2.7
Methanol	Barajin	616.1±4.6	283.9±4.1
	Zarabad	284.6±2.2	402.5±9.5
	Bahram abad	413.3±7.8	397.2±1.6
	Tehran	224.7±5.9	253.9±4.5
	Shahroud	348.9±3. 6	236.4±3.4
	Gorgan	72.7±1.5	30.1±3.5
	Ramsar	59.7±1.3	83.6±5.3
	Kashan	250.0±2.6	352.6±1.6
	Takab	216.6±5.6	253.9±1.7

* Barajin: *S. pinnatifida* subsp. *mucida*, Zarabad: *S. pinnatifida *subsp. *viridis*, Bahram abad: *S. pinnatifida* subsp. *viridis*, Pardis: *S. pinnatifida* subsp. *viridis*, Shahroud: *S. pinnatifida *subsp. *alpina*, Gorgan: *S. tournefortii*, Ramsar: *S. tournefortii*, Kashan:* S. tomentosa*, Takab:* S. persica*

**Table 3:** Cytotoxicity evaluation of different fractions of *Scutellaria* species

***Scutellaria*** ** samples**		**CC**_50 _ **± (SD) (µg/ml)** ^*^	**NCTC ± (SD) (µg/ml)** ^ **^	**Selectivity Index**
**Chloroform** **Fraction**	Barajin	37.07±(0.71)	18.50±(0.0)	2
	Zarabad	344.86±(58.43)	43.12±(0.0)	8
	Bahram abad	239.41±(0.90)	3.73±(0.0)	64.18
	Pardis	164.03±(0.62)	82.00±(0.0)	2
	Shahroud	159.419±(0.55)	79.50±(0.0)	2
	Gorgan	771.74±(2.89)	386.00±(0.0)	2
	Ramsar	92.97±(1.16)	23.25±(0.0)	4
	Kashan	169.01±(0.26)	2.64±(0.0)	64.02
	Takab	54.23±(0.49)	27.00±(0.0)	2
**Methanol** **Fraction**	Barajin	44.25±(1.33)	6.00±(0.0)	7.37
	Zarabad	225.04±(0.15)	14.06±(0.0)	16
	Bahram abad	5064.27±(1.27)	633.00±(0.0)	8
	Pardis	41.99±(2.15)	21.00±(0.0)	2
	Shahroud	80.19±(2.84)	40.00±(0.0)	2
	Gorgan	3.07±(0.19)	1.50±(0.0)	2
	Ramsar	6157.12±(1.02)	384.81±(0.0)	16
	Kashan	142.94±(8.68)	35.75±(0.0)	4
	Takab	141.97±(2.26)	97.00±(0.0)	1.46
**Amantadine hydrochloride**		197.01±(1.53)	98.50	2
**Oseltamivir carboxylate**		788.50±(6.01)	394.25	2

*CC_50_: Cytotoxic concentration 50 %; **NCTC: Non-cytotoxic concentration, Barajin: *S. pinnatifida* subsp. *mucida*, Zarabad: *S. pinnatifida* subsp. *viridis*, Bahram abad: *S. pinnatifida* subsp. *viridis*, Pardis: *S. pinnatifida *subsp. *viridis*, Shahroud: *S. pinnatifida* subsp. *alpina*, Gorgan: *S. tournefortii*, Ramsar: *S. tournefortii*, Kashan: *S. tomentosa*, Takab: *S. persica*

## Discussion

Medicinal plants have been used against various ailments, particularly infectious diseases. Medicinal plants have progressively been noticed as suitable alternatives to synthetic antiviral agents. 

One of the important effects of the *Scutellaria* genus has been the antiviral effect in East Asia, especially in Traditional Chinese Medicine (TCM) (29). *Scutellaria* species have different uses in traditional and folklore medicine. In this study, various Iranian endemic species of *Scutellaria* were compared based on the total phenol and flavonoid contents and their antiviral capacity against the IAV (H1N1). This genus was introduced as a source of flavonoids, so until 2010, out of 295 identified compounds, 164 belonged to the flavonoids class (11). Several literatures investigated the antiviral activity of some flavonoids such as baicalin, baicalein, wogonin, ganhuangenin, oroxylin A, scutellarin isolated from *Scutellaria* *baicalensis* (11). Flavonoids, as a secondary metabolite of plants, are found in all parts of the plants (root, stem, leaf, and flower). Flavonoids have shown protective potential against injuries, pathogens, and UV radiation (30). In humans, this class of phenolic compounds participates in anti-inflammatory, anti-allergic, anti-thrombotic, anti-diabetic, antiviral, antimicrobial, anti-carcinogenic, hepatoprotective, cardioprotective, neuroprotective, and anti-oxidant properties (21). Also, flavonoids have shown anti-platelet aggregation activities (31) and cancer-preventive effects (32). Flavonoids are composed of two pathways. The A ring is synthesized from the acetate pathway, including resorcinol or phloroglucinol molecules, and the B ring is synthesized from the shikimate pathway, including shikimic acid (33, 34). Flavonoids are potent anti-oxidants due to the presence of hydroxyl groups in the A and B rings, a 4-oxo functional group in the C ring, their ability to chelate divalent and trivalent cations, and changing the redox chemistry of chelating agents (35). The results of this study demonstrated that methanol fractions of different varieties of *S.* *pinnatifida* subsp. *viridis* have considerable amounts of phenols and flavonoids. Due to the polarity of flavonoids, they were more likely to present in methanol than in chloroform fraction. Each sample of *Scutellaria* species with a selectivity index of 2 or above, cell protection percentage of 40 % or above, and log HA decrement of 4.00 or above was considered a suitable candidate for the potential anti-influenza activity. The current study indicated that treatment of IAV with the selected extracts and compounds reduced the hemagglutination activity of the virus, which may result from the physical interaction of the extracts with the virus hemagglutinin. Five flavonoids were isolated and identified by different spectroscopic methods: luteolin and luteolin-7-*O*-glucoside, quercetin-3-*O*-glucoside, kaempferol-3-*O*-glucoside, and apigenin-4′-methoxy-7-*O*-glucoside.

Luteolin and luteolin derivatives are the most common flavonoids in edible plants such as *Daucus* *carota* L. (carrot), *Capsicum* *annuum* L. (peppers), *Apium* *graveolens* L. (celery), *Olea* *europaea* L. (olive), *Lactuca* *sativa* L. (lettuce), *Punica* *granatum* L. (pomegranate), *Theobroma* *cacao* L. (cacao), *Capparis* *spinosa* L. (capers), and *Cucumis* *sativus* L. (cucumber). Literature has attributed neuroprotective, anti-inflammatory, anti-diabetic, anti-oxidant, and anti-cancer effects to luteolin and luteolin derivatives (36). The addition of a double bond between carbon 2 and 3 in the C ring of eriodictyol by flavone synthase results in luteolin formation. Another pathway to luteolin formation is adding a hydroxyl group to apigenin by flavonoid 3′-hydroxylase (37). Luteolin can be absorbed in the small intestine and its metabolism pathway, including glucuronidation and sulfation, occurs in the liver (38). The excretion of luteolin in urine is very low. Therefore, the main route of luteolin elimination is via feces (39). The antiviral mechanism of luteolin is unclear yet. But, due to the structural similarity between quercetin and luteolin, the ability to binding to the S2 protein of coronavirus is expected (40).

Quercetin is another flavonoid that is present in *Scutellaria* genus. Quercetin and its derivatives showed several effects, such as anti-microbial, anti-cancer, anti-oxidant, and prevention of cardiovascular and immunomodulatory diseases (41, 42). Also, quercetin has demonstrated a considerable effect on SARS and MERS viruses, which has been suggested for treating the coronavirus known as COVID-19 (43). *Theobroma* *cacao* L. (cacao), *Vaccinium* *myrtillus* L. (cranberry),  *V. vitis-idaea *L. (lingonberry), *Brassica oleracea *L. (kale, broccoli),* Apium* *graveolens* L. (celery), *Lactuca* *sativa* L. (lettuce), *Solanum lycopersicum *L. (tomato), *Daucus* *carota* L. (carrot), and *Ginkgo biloba* L. (Maidenhair tree) are foods rich in quercetin (44). This flavonoid is formed from two biosynthesis pathways. In the first pathway, kaempferol is formed as an intermediate compound. A hydroxyl group is added to kaempferol by kaempferol 3′-monooxygenase enzyme and 3′,4′-dihydroxy kaempferol is formed as quercetin. In the second pathway, dihydrokaempferol 3′-hydroxylase synthesizes taxifolin. Then, taxifolin is converted to quercetin by quercetin synthase (45). Research has shown the microflora of the large intestine can cleave flavonoids into phenylpropionic acid, phenylacetic acid, and some other compounds (46). According to previous studies, quercetin is eliminated through feces as aglycone (42). One possible mechanism of quercetin has been found to be the inhibition of mRNA synthesis and M2 protein expression in IAV infection (47).

Astragalin (kaempferol-3-*O*-glucoside) is widely distributed in plants. The source of kaempferol and its derivatives in edible plants is *Allium cepa *L. (onion),* Brassica oleracea *L. (broccoli),* Petroselinum crispum *(Mill.) Fuss. (parsley),* Lactuca sativa *L. (lettuce),* Spinacia oleracea *L. (spinach), *Rubus idaeus *L. (raspberry),* Fragaria vesca* L. (strawberry), *Malus domestica *Borkh*. *(apple),* Solanum lycopersicum *L. (tomato),* Citrus paradisi *Macfad. (grapefruit), *Pistacia vera *L. (pistachio), and *Vitis vinifera *L. (grape). (48)*. Kae*mpferol and its derivatives showed anti-oxidant, anti-inflammatory, anti-cancer, anti-diabetic, cardioprotective, and neuroprotective effects (48, 49). Recent studies demonstrated that kaempferol and quercetin might execute beneficial effects on the treatment or control of coronavirus (COVID-19) by inhibiting and phosphorylating protein kinase A (50). In the biosynthesis pathway of kaempferol, flavonone-3-dioxygenase enzyme adds a hydroxyl group to C3, and dihydrokaempferol is formed. Then, flavone synthase enzyme forms a double bond in C2-C3 position to produce kaempferol. Adding a glucose molecule by flavonol-3-*O*-glucosyltrasferase led to the formation of astragalin (51). Kaempferol (IC_50_= 0.5 μM) was a potent anti-oxidant by scavenging free radicals and reducing the hydroxyl ions (52, 53). Kaempferol and its derivatives are absorbed in the small intestine by facilitated or passive diffusion or active transport. The kaempferol is metabolized by glucuronidation in the liver and colon microflora and its excretion occurs through urine (54).

Acacetin and other apigenin derivatives are abundant in *Achillea millefolium* L. (Yarrow), *Apium* *graveolens* L. (Celery), *Artemisia* *dracunculus* L. (Tarragon), *Coriandrum* *sativum* L. (Cilantro), *Digitalis* *purpurea* L. (Purple foxglove), *Gingko* *biloba* (Maidenhair tree), *Glycyrrhiza* *glabra* L. (Licorice), *Linum* *usitatissimum* L. (flax), *Matricaria* *chamomilla* L. (chamomile), *Mentha* *spicata* L. (Spearmint), *Ocimum* *basilicum* L. (Basil), and *Origanum* *vulgare* L. (Oregano) (55). Apigenin and its derivatives have shown anti-oxidant, anti-cancer, anti-inflammatory, anti-mutagenic, hepatoprotective, anti-microbial, and neuroprotective effects (44, 56). Apigenin 4′-*O*-methyltransferase synthesizes 4′-methoxy apigenin, known as acacetin. This flavonoid is isolated from *Chrysanthemum morifolium *for the first time (57). Apigenin is absorbed in the small intestine and is metabolized by glucuronidation and sulfation in liver tissue (58). The elimination of apigenin is through urine and feces (59). A study showed the antiviral effect of apigenin on influenza virus type A, which protected cells from death and suppressed replication of IAV by inhibiting the neuraminidase of the virus (60).

## Conclusion

Based on this scientific confirmation, the Methanol fraction of *S. pinnatifida* subsp. *viridis* and methanol fraction of *S. tomentosa *that showed the best antiviral activity might have the capacity to ease the symptoms and burden of the flu infection. Recommendations are proposed for strategizing the future role and place for medicinal plants against influenza infection and other infectious diseases prevention and therapy in national, regional, and international health policies and programs. Antiviral effects of *Scutellaria* species and also isolation and identification of some flavonoids such as apigenin, kaempferol, quercetin, and luteolin and their derivatives from Iranian species of *Scutellaria, *introduced this genus, especially *S. pinnatifida* due to wide geographical distribution in Iran, as a potent candidate for the treatment of influenza A virus/H1N1. Also, these flavonoids can be used as supplements to prevent viral diseases such as influenza, and COVID-19. For the first time, anti-influenza virus effect of some Iranian species of *Scutellaria* was investigated and these flavonoids (kaempferol, apigenin, quercetin, and luteolin) were isolated from *Scutellaria* *pinnatifida* subsp. *viridis*. The next focus of this study will be the purification of pure compounds responsible for the bioactivity against IAV infection and their mode of action. As well, *S. pinnatifida* can be introduced as a new source for treating or preventing influenza virus as an alternative medicine or in combination with other anti-influenza drugs.

## Authors’ Contributions

ZT, and PM Study conception and design; MPH, and MAK Data processing, collection, perform experiment; MPH, and HSh Analysis and interpretation of results; MPH, MAK, HSh Draft manuscript preparation; SG, MShA, AH, MPH, and MHGh Critical revision or editing of the article; ZT, and PM Final approval of the version to be published; ZT, and SG Supervision funding acquisition.

## Conflicts of Interest

The authors declare that there is no conflict of interest.
